# A journey mapping study of internship stress among undergraduate nursing interns

**DOI:** 10.3389/fpubh.2026.1958747

**Published:** 2026-10-28

**Authors:** Yuhang Cheng, Rong Zeng, Yi Li, Yan Huang, Huilan Wang, Yuzi Zhao, Wenxiu Jiang, Qian Yang

**Affiliations:** 1 Chengdu Medical College School of Nursing, Chengdu, Sichuan, China; 2 The First Affiliated Hospital of Chengdu Medical College, Chengdu, Sichuan, China; 3 Sichuan College of Traditional Chinese Medicine, Deyang, Sichuan, China

**Keywords:** health, journey mapping, nursing education, stress, undergraduate nursing interns

## Abstract

**Aim:**

To explore the dynamic process of stress experienced by undergraduate nursing interns during clinical internships and to construct a journey map of internship stress in order to identify stage-specific stressors, emotional experiences, and pain points.

**Methods:**

Using purposive sampling, 19 undergraduate nursing interns were recruited from five tertiary hospitals in Sichuan Province between May 2025 and March 2026. Data were collected at different stages of the internship through semi-structured interviews. Thematic analysis was employed for coding, and journey mapping was applied to integrate and visually represent the trajectory of stress over time.

**Results:**

Internship-related stress among undergraduate nursing interns evolved across four periods: Preparation Period; Shock Period; Adaptation Period; Differentiation Period. A total of 36 themes were identified across three overarching dimensions—tasks, emotional experiences, and pain points—systematically illustrating the trajectory of stress evolution.

**Conclusion:**

Stress among undergraduate nursing interns demonstrates a stage-based and dynamic trajectory, with distinct pain points and corresponding support needs at each stage. These findings provide empirical evidence for nursing educators and clinical managers to develop stage-specific, targeted interventions and to optimize internship support systems.

## 1 Background

Clinical internship is a critical stage in which nursing students translate theoretical knowledge into practical competence while developing clinical thinking and professional attributes. However, during this period, undergraduate nursing interns are required not only to perform clinical skills and adapt to the healthcare environment, but also to manage multiple demands, including nurse–patient communication and career planning, which collectively impose considerable psychological stress (1). Such stress may not only compromise internship performance but also contribute to burnout, anxiety, and depression, and may even undermine professional commitment, ultimately leading to attrition in the nursing workforce (2). Therefore, a comprehensive understanding of the development and progression of internship-related stress among undergraduate nursing interns is of significant importance for optimizing nursing education.

From a theoretical perspective, clinical internship represents not merely a process of skill acquisition, but also a critical transitional phase in which students shift from the role of learner to that of professional nurse. According to Transition Theory proposed by Meleis et al. (3), individuals undergoing role transitions often experience identity reconstruction, competence adaptation, and emotional fluctuations. This process is influenced by multiple factors, including personal characteristics, environmental support, and interpersonal interactions, and is inherently dynamic. Within this framework, the stress experienced by undergraduate nursing interns should not be viewed as static, but rather as embedded within the transition process and continuously evolving over time. Importantly, interns’ responses to internship stress are not shaped solely by external stressors. Individual psychological resources including emotional regulation, self-efficacy, and resilience act as key moderators of stress perception and role adaptation (4), which helps account for divergent adaptation trajectories among interns in similar clinical settings.

However, existing studies have predominantly focused on the status quo or influencing factors of stress, largely employing cross-sectional designs that capture stress at a single time point. Existing research on nursing internship stress falls into three broad strands: prevalence studies documenting the overall level of stress and associated mental health outcomes, studies identifying static stressors and predictive factors, and a small number of longitudinal or qualitative studies focusing on early transition shock or end-of-internship career outcomes (5–7). There remains a lack of systematic investigation into the trajectory of stress throughout the entire internship, as well as the identification of critical influencing points across different stages. In particular, the mechanisms through which stress shifts from one stage to another, and the stage-specific pain points and support needs corresponding to each phase, remain largely underexplored.

Journey mapping, as a process-oriented research approach, emphasizes the temporal sequence of experiences and facilitates the identification of key touchpoints, emotional changes, pain points, and needs across different stage (8). It enables an intuitive visualization of complex and evolving experiences. In recent years, journey mapping has been increasingly applied in healthcare services, patient experience, and educational research, offering a novel perspective for understanding individuals’ continuous experiences within specific contexts (9–11). The application of journey map to the study of internship stress among undergraduate nursing interns may enable a comprehensive depiction of stress development and evolution from an integrated perspective of stages, events, emotions, and needs within the framework of Transition Theory.

Therefore, guided by Transition Theory, which serves as the overarching conceptual framework informing study design, interview guide development, thematic coding, and interpretation of findings, this study adopted a longitudinal qualitative design and employed journey mapping to illustrate the trajectory of internship-related stress among undergraduate nursing interns. It aimed to identify stage-specific core tasks, emotional characteristics, and key pain points, thereby providing theoretical evidence and practical implications for nursing educators and clinical managers to optimize internship support systems and implement stage-specific targeted interventions.

## 2 Methods

### 2.1 Participants

Purposive sampling was used to recruit undergraduate nursing interns undertaking clinical placements in five tertiary hospitals in Sichuan Province between May 2025 and March 2026.

#### 2.1.1 Inclusion criteria

Participants were eligible if they: (1) were full-time undergraduate nursing students about to commence their compulsory clinical internship; (2) were enrolled in a clinical internship program of at least 10 months in duration, ensuring coverage of all four study stages; and (3) provided informed consent and agreed to participate voluntarily.

#### 2.1.2 Exclusion criteria

Participants were excluded if they: (1) had interrupted their internship due to personal or medical leave; or (2) had prior internship experience.

#### 2.1.3 Sample size

Sample size was determined by data saturation, defined as the point at which no new themes emerged. A total of 19 undergraduate nursing interns were included and assigned identification numbers (N1–N19).

### 2.2 Ethical approval

Ethical approval was obtained from the Ethics Committee of Chengdu Medical College (Approval No. 2025NO.044). The study adhered to the principles of the Declaration of Helsinki. Written informed consent was obtained from all participants, and voluntary participation was ensured.

### 2.3 Study design

A prospective longitudinal qualitative design was adopted to systematically track the dynamic trajectory of internship-related stress among the same cohort across different stages of their clinical placement. Rather than using fixed-interval follow-ups, this study employed a stage-driven, event-based sampling schedule aligned with Meleis’ Transition Theory and the core principles of journey mapping methodology, to ensure targeted capture of stress experiences at critical role transition touchpoints. Following thematic analysis, the journey map was used as a visual and organizational framework to integrate and present the findings (11), with identified themes mapped onto different stages of the internship. This study was reported in accordance with the Consolidated Criteria for Reporting Qualitative Research (COREQ) guidelines to ensure methodological transparency and rigor.

### 2.4 Development of the interview guide

The research team systematically searched literature related to stress among nursing interns in CNKI, PubMed, and other databases, and screened relevant articles published in the past 5 years. Based on the research objectives, combined with expert consultation and internal discussions within the research team, a preliminary interview guide was developed. Concurrently, to ensure clear question wording, logical sequencing, and practical feasibility, pilot interviews were first conducted with two nursing interns not included in the formal sample. Based on the feedback from the pilot interviews, the question sequence was optimized and redundant expressions were simplified to elicit more accurate descriptions of internship stress. The final version of the interview guide comprises a total of 16 questions. The semi-structured interview guide used in this study was developed exclusively by the research team for this project and has not been published elsewhere previously. The English version of the final interview guide is provided as Supplementary File 1 and is cited herein for reference.

### 2.5 Data collection

Prior to data collection, participants were recruited through official study announcements distributed to undergraduate nursing interns who were about to commence their clinical placement via the faculty’s internship coordinators. For students who expressed interest and met the study’s inclusion criteria, the research team conducted one-on-one consultations to provide a comprehensive explanation of the study purpose, interview schedule, required time commitment, and data confidentiality protocols. Formal interviews were arranged only after participants confirmed their willingness to participate (see Table 1).

**Table 1:** Demographic and clinical characteristics of participants (*n* = 19).

ID	Age	Gender	Affiliated teaching hospital	Only child	Family healthcare background	Career intention
N1	20	Female	Hospital A	No	Yes	Clinical nursing practice
N2	21	Female	Hospital E	Yes	No	Clinical nursing practice
N3	21	Female	Hospital C	No	No	Clinical nursing practice
N4	22	Male	Hospital E	Yes	Yes	Nursing education
N5	21	Female	Hospital C	No	Yes	Clinical nursing practice
N6	20	Female	Hospital A	Yes	No	Undecided
N7	22	Female	Hospital A	No	No	Postgraduate study
N8	21	Male	Hospital C	Yes	Yes	Clinical nursing practice
N9	22	Female	Hospital B	Yes	Yes	Postgraduate study
N10	20	Female	Hospital E	Yes	No	Clinical nursing practice
N11	21	Female	Hospital A	Yes	No	Clinical nursing practice
N12	22	Female	Hospital E	Yes	Yes	Postgraduate study
N13	23	Male	Hospital E	No	No	Postgraduate study
N14	21	Female	Hospital A	Yes	Yes	Undecided
N15	21	Female	Hospital D	No	No	Nursing education
N16	22	Female	Hospital C	No	Yes	Postgraduate study
N17	21	Female	Hospital A	No	No	Clinical nursing practice
N18	21	Male	Hospital D	Yes	No	Clinical nursing practice
N19	23	Female	Hospital B	Yes	Yes	Undecided

Hospital A: The First Affiliated Hospital of Chengdu Medical College. Hospital B: Mianyang People’s Hospital. Hospital C: The General Hospital of Western Theater Command. Hospital D: The FirstPeople’s Hospital of Yibin. Hospital E: The Second Affiliated Hospital of Chengdu Medical College.

Interviews were conducted in a quiet, private meeting room. Prior to each interview, the researcher explained the study purpose and procedures in detail to ensure informed and voluntary participation. Written informed consent and confidentiality agreements were obtained from all participants.

Semi-structured interviews were conducted by trained qualitative researchers, with each interview lasting approximately 20–40 min. All interviews were audio-recorded with participants’ permission. To enhance the credibility, accuracy, and richness of the data, interviewers employed techniques such as probing, paraphrasing, and clarification to verify participants’ perspectives and experiences. The order and phrasing of questions were flexibly adjusted to maintain a natural conversational flow. Data collection continued until thematic saturation was achieved.

Interviews were prospectively scheduled at four predefined stage-aligned time points, with each participant completing one semi-structured interview per stage. All interviews were conducted in real time at the corresponding phase of internship to minimize recall bias and ensure the authenticity of stage-specific experiences. A total of 76 interviews were completed with all 19 participants, yielding a 100% retention and completion rate across all time points. The specific timing, corresponding internship stage, and core focus of each interview wave are detailed in Table 2.

**Table 2:** Timing of interviews across internship stages.

Time point	Corresponding internship stage	Interview timing	Number of participants	Interview focus
T0	Preparation period	1 week before the official start of clinical internship	*N* = 19	Pre-internship expectations, self-assessment of preparedness, and anticipated stressors
T1	Shock period	4 weeks after the start of clinical internship	*N* = 19	Initial clinical adaptation, early procedural stressors, and emotional responses to role transition
T2	Adaptation period	20 weeks after the start of clinical internship	*N* = 19	Skill proficiency, cross-department rotation adaptation, multi-task conflicts, and changes in perceived stress intensity
T3	Differentiation period	36 weeks after the start of clinical internship	*N* = 19	Career decision-making, professional identity formation, burnout, and end-of-internship stressors

### 2.6 Data analysis and rigour

Data were analysed using reflexive thematic analysis as described by Braun and Clarke (12) from a constructivist epistemology. All interviews were transcribed verbatim within 24–48 h and cross-checked for accuracy. The analysis followed six standard steps: data familiarization, initial coding, category development, theme generation, theme review, and theme definition.

Dual independent coding was implemented as a reflexivity and quality control strategy, not as a quantitative inter-coder reliability test typical of codebook-based analysis. Two researchers coded the dataset independently and resolved discrepancies through discussion to minimize individual bias and integrate multiple interpretive perspectives. A practice aligned with the core principle of reflexive thematic analysis, which emphasizes active and critical engagement of researchers in meaning-making rather than pursuit of numerical reliability metrics. No pre-specified codebook was used at any stage of the analysis, and an audit trail was maintained throughout the process.

A hybrid inductive-deductive coding approach was adopted, with Transition Theory as the overarching conceptual anchor. Deductively, the theory informed study design, interview guide development, and initial coding sensitivity to core transitional processes (role adaptation, identity reconstruction, emotional fluctuation). Inductively, all themes emerged bottom-up from the interview data, with coders open to unanticipated findings beyond the theoretical framework. Transition Theory was further applied to interpret and integrate emergent themes into the journey map structure. The frequency of thematic mentions was used as a descriptive indicator to assist in judging the prevalence and salience of themes, alongside depth of meaning and clinical relevance, rather than serving as a formal quantitative test of significance.

Given the longitudinal design, a two-stage analytical strategy was employed: Within-case analysis: Full stress trajectories across all four stages were mapped for each of the 19 participants to preserve idiosyncratic transition patterns and longitudinal integrity. Cross-case analysis: Systematic comparison across individual trajectories was conducted to identify shared stage-specific themes, common patterns, and subgroup variations. The final journey map integrated cross-case findings, grounded in individual longitudinal experience rather than aggregated stage-based quotations.

Identified themes were mapped onto internship stages to construct the internship stress journey map, which served as both a data presentation tool and an integrative analytical framework. Trustworthiness was ensured via member checking, ongoing team discussion, and reflexive journaling.

For the stress trajectory curve embedded in the journey map, the full interview transcript of each participant at each stage served as the unit of analysis (76 units in total, 19 participants × 4 stages). The relative stress intensity score was calculated using an “intensity rating × normalized frequency” approach: the frequency of stress-related statements was normalized per 1,000 characters of transcript to control for confounding from varying interview duration; two independent researchers then rated perceived stress intensity on a 3-point ordinal scale (1 = low, 2 = moderate, 3 = high). The product of these two metrics generated individual scores for each stage. After resolving coding and rating discrepancies through team discussion, the median score across all 19 participants at each stage was adopted as the group-level relative stress value. Repeated mentions of the same stressor within a stage were retained in the frequency count to reflect its psychological salience. The resulting curve reflects relative changes in stress level across stages rather than absolute quantitative values; the two branching lines in the Differentiation Period are a qualitative representation of diverging stress trajectories driven by professional identity polarization, not precise quantitative grouping.

## 3 Results

A total of 76 interviews were conducted with 19 participants, all of whom completed the full study.

### 3.1 Development of the journey map of internship-related stress

A journey map typically consists of a horizontal axis (timeline) and a vertical axis (task dimension) (13). Guided by Transition Theory (3), and informed by literature review and practical experience, the research team delineated four stages along the timeline: Preparation Period (2–4 weeks before internship), Shock Period (first month), Adaptation Period (months 2–7), and Differentiation Period (months 8–10).

The vertical axis comprised three dimensions aligned with the study aims: tasks, emotional experiences, and pain points. Based on interview data, factors influencing stress and their trajectories were analysed across stages.

#### 3.1.1 Preparation period

##### 3.1.1.1 Tasks

(1) Pre-adaptation of the student–intern role. Undergraduate nursing interns need to psychologically complete the expected transition from being a student to becoming an intern.
  *N1*: “From being a complete beginner to entering the internship stage, I feel that I cannot make a very good transition.”
(2) Assessment of skills and knowledge preparation. Undergraduate nursing interns need to conduct self-assessment of the theoretical knowledge and operational skills they have mastered, and identify the gap between themselves and clinical requirements.
  *N1*: “I’m afraid of making mistakes in operations and worry that my ability is not enough to meet clinical requirements.” “I cannot keep up with what the teachers teach.”
  *N13*: “What I worry about most is the issue of operational skills.”
(3) Advance planning of internship and personal development. Some undergraduate nursing interns plan their time and energy in advance, coordinating internship tasks with personal development matters such as postgraduate entrance exams and graduation.
  *N9*: “Before the internship even started, I was already thinking about how to prepare for the postgraduate entrance exam and write my thesis.”

##### 3.1.1.2 Emotions

(1) Anxiety. Uncertainty about the clinical environment, practical skills, and future development leads to widespread psychological anxiety among undergraduate nursing interns.
  *N2*: “I’m a bit afraid of the clinical setting and scared of performing invasive procedures on patients.”
  *N7*: “I’m unfamiliar with the internship environment and worried that teachers will be strict.”
(2) Expectation. The novelty of leaving classroom learning and entering clinical practice generates positive expectations toward internship life.
  *N3*: “I do not need to attend classes anymore, and I feel excited about entering clinical practice.”

##### 3.1.1.3 Pain points

(1) Information asymmetry. Undergraduate nursing interns lack concrete understanding of real clinical work and find it difficult to predict potential difficulties.
  *N8*: “I do not understand the Hospital And internship situation, and I worry that I will not be able to adapt and do not know what problems I will encounter.”
(2) Self-doubt. Undergraduate nursing interns lack confidence in their theoretical knowledge and operational skills and worry about being unable to perform clinical work.
  *N1*: “There is a gap between my theory and practice, and I’m afraid I cannot keep up.”
  *N2*: “I’m afraid that I will not be able to do anything well during the internship.”
(3) Early accumulation of multiple pressures. Before the internship formally begins, multiple pressures such as postgraduate exams and thesis writing have already emerged.
  *N5*: “I have to do the internship and look for a job at the same time, which is very stressful.”
  *N16*: “The internship conflicts with preparing for postgraduate exams, and I also have to write my thesis—I really cannot handle it.”

#### 3.1.2 Shock period

##### 3.1.2.1 Tasks

(1) Adapting to the clinical environment. Undergraduate nursing interns need to quickly become familiar with ward layout, workflows, and regulations.
  *N13*: “When I first entered the clinical setting, I did not understand anything about the department and did not know what to do.”
(2) Learning basic operational skills. Undergraduate nursing interns begin clinical practice and attempt to perform basic nursing procedures such as venipuncture and catheterization.
  *N1*: “I know it in theory, but in actual operation there are many details and I lack clinical experience.”
  *N2*: “When performing the first invasive procedure, I was very panicked, and my hands were shaking.”

##### 3.1.2.2 Emotions

(1) Panic and nervousness during practice. When performing procedures for the first time, undergraduate nursing interns experience trembling hands, fear of failure, and helplessness when facing patient refusal.
  *N1*: “Many times I do not know what to do and feel very panicked.”
  *N6*: “At the beginning, I was really scared and did not dare to do it.”
(2) Frustration and self-denial. After failures, patient rejection, or criticism from instructors, undergraduate nursing interns question their professional suitability.
  *N3*: “When patients do not let you do it, you feel like maybe you are not capable.”
  *N18*: “After being criticized, I would doubt whether I am suitable.”

##### 3.1.2.3 Pain points

(1) Insufficient operational skills. Although undergraduate nursing interns have theoretical knowledge, they cannot translate it into fluent and accurate clinical practice.
  *N1*: “When actually doing it, I still cannot. It feels like my mind knows it, but my hands do not.”
  *N14*: “My skills are not proficient, and I’m afraid of being blamed by teachers.”
(2) Lack of nurse–patient trust. Patients resist undergraduate nursing interns’ identity and directly refuse procedures, reducing opportunities for skill practice.
  *N3*: “As soon as you approach, patients know you are an intern and say they do not want an intern and ask for the teacher.”
  *N15*: “Some patients worry we will not do it well, so they will not let us do it and will call the teacher instead.”
(3) Disconnection between theory and practice. Differences between textbook standards and clinical practice make decision-making difficult.
  *N2*: “I do not know whether to follow theory or clinical practice.”
  N5: “What you learn in books is rarely used in real clinical work.”
(4) Difficulty adapting to night shifts. Undergraduate nursing interns struggle with night shifts, leading to sleep deprivation and physical and mental fatigue.
  *N5*: “Night shifts are the most stressful.”
  *N10*: “Sleep is clearly insufficient, and I feel exhausted.”

#### 3.1.3 Adaptation period

##### 3.1.3.1 Tasks

(1) Proficient mastery of nursing operations. Most undergraduate nursing interns transition from assisted operations to independent completion, acquiring basic clinical nursing competence.
  *N11*: “Later, I could complete some procedures on my own.”
  *N19*: “I can independently take care of one patient bed.”
(2) Dynamic adaptation across multiple departments. Undergraduate nursing interns adjust their working modes according to different departmental characteristics and adapt to varying clinical environments.
  *N8*: “Each department is different, and you need to adapt again.”
Balancing multiple tasks and time management. Undergraduate nursing interns coordinate internship, academic tasks, and postgraduate preparation, compressing personal time to manage multiple responsibilities.
  *N13*: “I need to do the internship while arranging my thesis.”
  *N15*: “Job interviews require asking teachers for leave.”
(3) Clinical communication and interpersonal adaptation. Undergraduate nursing interns learn how to communicate with supervisors and patients and adapt to departmental interpersonal environments.
  *N13*: “Different departments have different communication styles, and you need to gradually adapt to their pace.”
  *N17*: “You need to communicate with patients and coordinate time with teachers, and sometimes pay attention to teachers’ attitudes.”

##### 3.1.3.2 Emotions

(1) Sense of imbalance. Lack of payment, unequal workload, and differences among peers lead to psychological imbalance.
  *N1*: “I feel like I’m paying to work, which feels unfair. Some classmates do less and leave earlier, and comparing makes it worse.”
(2) Emerging burnout. Repetitive basic nursing tasks and high workload reduce motivation.
  *N1*: “In the later stage, the skills I’ve learned become repetitive, and my motivation decreases.”
  *N2*: “There is a sense of burnout later, and I’m not as motivated as before.”
(3) Emotional stabilization. After the intense stress of the shock stage, undergraduate nursing interns gradually adapt to the clinical rhythm, with reduced emotional fluctuation and lower intensity of negative emotions.
  *N12*: “Later, I could balance things better, and the pressure was less than before.”
  *N10*: “My mindset became calmer.”
(4) Increased sense of achievement. Completing tasks independently and receiving recognition enhances professional confidence and competence.
  *N11*: “Completing a procedure gives me a sense of achievement.”
  *N18*: “When an elderly patient gave me a thumbs-up during blood collection, I felt I was capable.”

##### 3.1.3.3 Pain points

(1) Departments and supervising teachers vary greatly. Variations in workload and teaching styles directly affect stress experiences.
  *N1*: “In busy departments, I worry about not learning everything; in less busy ones, I worry about not learning anything.”
  *N5*: “When encountering arrogant teachers, I feel annoyed and resist them.”
(2) Conflicts among multiple tasks. Internship schedules overlap with postgraduate exams, thesis writing, and job seeking, forming a major stress source.
  *N13*: “Postgraduate exams, job search, and thesis all come at once.”
  *N17*: “Interviews, night shifts, and thesis writing overlap.”
(3) Fatigue from repetitive work. Long-term repetitive basic nursing work with limited skill growth leads to sustained exhaustion.
  N16: “The work is the same every day. I already know how to do it, but it’s tiring and there’s no reward.”

#### 3.1.4 Differentiation period

##### 3.1.4.1 Tasks

(1) Completion of internship assessment and summary. Undergraduate nursing interns organise internship records and prepare for departmental and final assessments to ensure completion of internship requirements.
  *N9*: “I worry that some parts of my internship manual are not well completed and may cause problems when returning to school.”
  *N8*: “I’m afraid that some shortcomings during the internship may make teachers dissatisfied and they will not sign off.”
(2) Career decision-making and planning. Based on internship experiences and personal conditions, undergraduate nursing interns determine future paths such as employment, postgraduate study, or residency training.
  *N17*: “I’m not planning to take postgraduate exams. I will work after graduation or apply for standardized training.”
(3) Reflection on professional role. Undergraduate nursing interns review their internship experience and evaluate their suitability for the nursing profession.
  *N14*: “I keep thinking whether I am a qualified nurse and whether I can take on the responsibilities of a formal or training nurse.”
  *N19*: “The biggest value of the internship is deciding whether to continue in this profession.”

##### 3.1.4.2 Emotions

(1) Dual anxiety about employment and further study. Uncertainty about job outcomes and postgraduate results leads to strong anxiety.
  *N1*: “Anxiety about employment gradually increases in the later stage.”
  *N16*: “Employment anxiety exists, especially after March when the school pushes hard.”
(2) Polarization of professional identity. Positive experiences strengthen professional identity, while negative ones increase detachment, showing a polarized pattern.
  *N7*: “After the internship, I decided to change careers and never engage in clinical nursing again.”
  *N11*: “Being able to independently manage patients in ICU makes me feel capable.”
(3) Solidification of burnout. Long-term low reward and high workload lead to fixed burnout in some undergraduate nursing interns.
  *N3*: “Later, I just want to slack off at work and do not want to do anything.”
  *N16*: “I just want the internship to end and leave the hospital. I feel no energy or motivation.”

##### 3.1.4.3 Pain points

(1) Job and school pressure. Job search failures and uncertain postgraduate results become the main source of peak stress.
  *N17*: “Repeated interview failures create huge pressure.”
  *N7*: “From January to March is the peak stress period due to uncertainty of exam and interview results.”
(2) Loss of sense of professional meaning. Repetitive work and low financial return lead to questioning of professional value.
  *N3*: “The workload and salary do not match, so I do not want to continue nursing.”
  *N5*: “The effort and return are not proportional; it’s exhausting both physically and mentally.”
(3) Failure of support systems. Family and schools fail to provide empathetic support, leaving undergraduate nursing interns feeling isolated.
  *N7*: “Seeking help from parents does not really help.”
  *N12*: “When I talk to my parents, they cannot truly understand or empathize.”

### 3.2 Construction of the internship stress journey map for undergraduate nursing interns

Based on the principles of journey map construction, this study divided the timeline (horizontal axis) of internship stress among undergraduate nursing interns into four stages: the preparation stage, shock stage, adaptation stage, and differentiation stage. The vertical axis covers three dimensions during the internship process: tasks, emotional experiences, and stress pain points. Through coding and analysis of in-depth interview data from 19 undergraduate nursing interns, a total of 36 themes were extracted, and an internship stress journey map was constructed (Figure 1).

**Figure 1:**
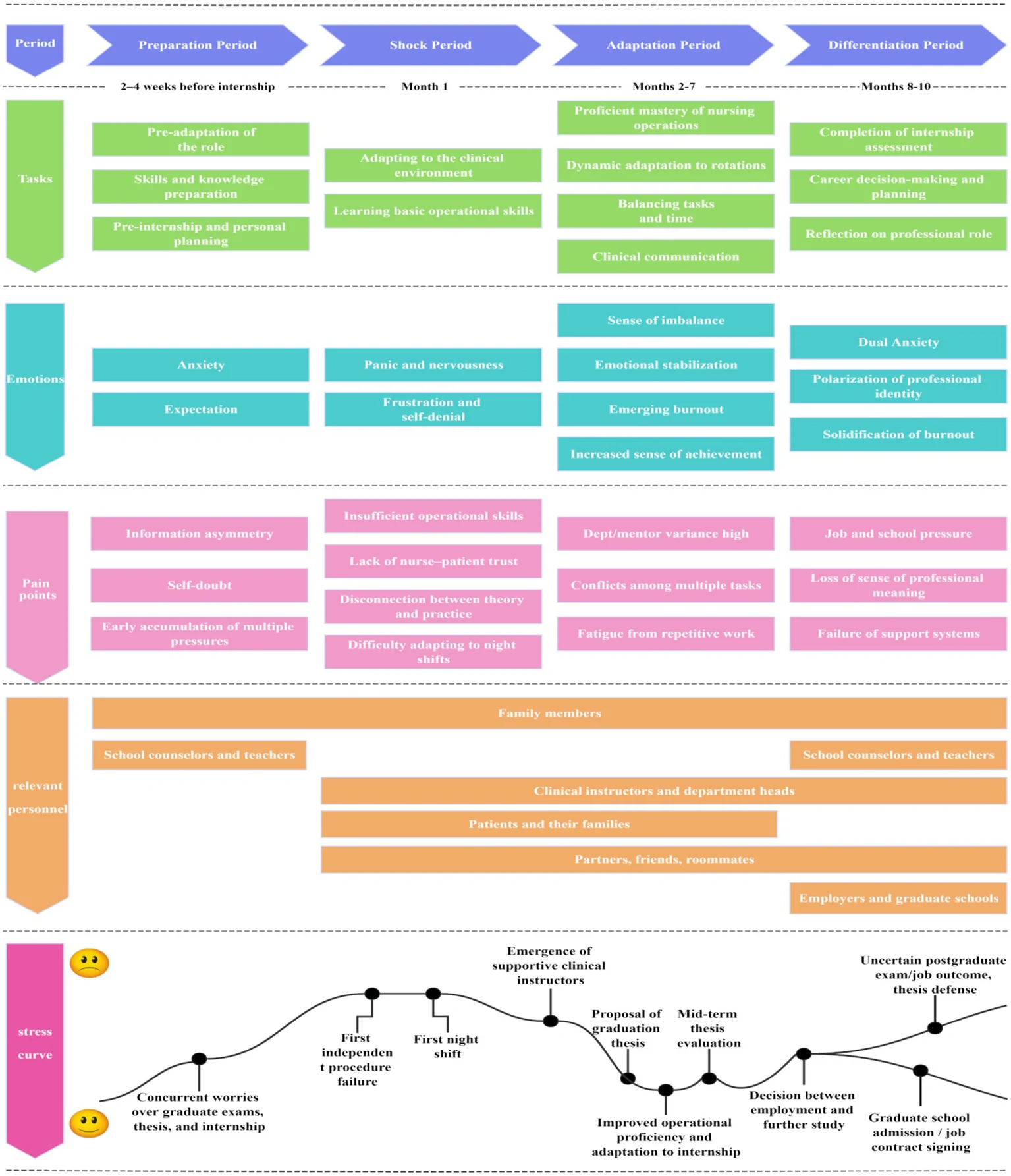
The internship stress journey map for undergraduate nursing interns. The stress trajectory curve is a qualitative schematic representation drawn based on stage-specific critical events and relative stress intensity, and does not represent continuous quantitative measurement.

## 4 Discussion

### 4.1 Establishing a proactive pre-internship support system

This study found that internship-related stress among undergraduate nursing interns does not originate from entering the clinical setting, but emerges significantly during the pre-internship preparation stage. From the perspective of Meleis’ Transition Theory, this pre-internship phase corresponds to the anticipatory phase of role transition, where the awareness property of transition first emerges. The information asymmetry identified in this stage, defined as a lack of concrete understanding of clinical workflows, role expectations and ward realities, reflects low situational awareness among interns prior to entering the clinical setting, which acts as a key antecedent of subsequent transition stress. This finding extends beyond previous research that primarily focuses on stressors during the internship period, and identifies the pre-internship phase as the first critical window for role transition.

In current nursing education practice, pre-internship training mainly emphasizes skills enhancement and institutional regulations, while insufficient attention is given to psychological preparation and cognitive readiness for the transition from student to clinical intern (2). In this study, most undergraduate nursing interns had already experienced stress prior to the internship, particularly related to the clinical environment, fear of procedural errors, and nurse–patient communication. Information asymmetry was identified as a primary trigger of stress (14). Undergraduate nursing interns often lack concrete understanding of real clinical scenarios, departmental workflows, and interaction patterns with patients, which leads to uncertainty, fear of the unknown, and self-doubt regarding their competence.

Furthermore, this study revealed that, unlike in many international nursing education contexts, undergraduate nursing interns in China commonly face simultaneous pressures during the preparation stage, including postgraduate entrance examinations, civil service exams, thesis writing, and job searching. However, current educational systems rarely provide proactive guidance on time management and multi-task coordination (15).

These findings suggest that nursing schools and hospitals may consider shifting the intervention point forward from the beginning of clinical placement to the pre-internship stage (16). On one hand, strategies such as clinical simulation, experience-sharing from senior interns, and comprehensive introductions to departmental workflows may help reduce information gaps and alleviate anticipatory stress. On the other hand, preparatory courses on multi-task management and coordinated scheduling mechanisms could be explored to facilitate role pre-adaptation and support a smoother transition into the shock stage of the internship.

### 4.2 Developing standardized onboarding and clinical teaching systems

The findings indicate that the shock stage represents the first peak of stress during the internship, characterized by acute psychological impact from entering the clinical environment and role transition. Consistent with Meleis’ Transition Theory, this acute stress profile directly aligns with the concept of role insufficiency. The information asymmetry that originates in the preparation period persists into the early clinical phase, and when coupled with the gap between theoretical knowledge and practical competence, it leaves interns unable to fully meet the demands of the new clinical role, which forms the core mechanism driving the first stress peak. Core emotional responses include procedural anxiety, frustration, and self-doubt, while key stressors involve insufficient clinical skills, lack of nurse–patient trust, discrepancies between theory and practice, and difficulties adapting to night shifts. These stressors are primarily task-related and skill-centered.

Previous studies have shown that the early stage of clinical practice is a high-risk period for burnout and weakened professional identity among undergraduate nursing interns (17). However, few studies have longitudinally examined the mechanisms underlying stress formation at this stage. This study, through a journey mapping approach, suggests that the core issue is not merely insufficient technical proficiency, but a systemic barrier in translating theoretical knowledge into clinical practice (18).

First, discrepancies between simulated training environments and real clinical settings result in a gap between knowing and doing. Second, patient resistance to undergraduate nursing interns involvement reduces opportunities for hands-on practice (19). Additionally, the study highlights the impact of night shift adaptation on interns’ physical and psychological well-being. The commonly observed lack of graded night shift arrangements further exacerbates fatigue and stress (20).

To address these stage-specific challenges, hospitals may consider establishing standardized onboarding and teaching systems, including stepwise training models that gradually increase autonomy in clinical tasks. Simulation-based communication training and patient education initiatives may help enhance nurse–patient interaction skills and improve public understanding of supervised clinical training, thereby reducing trust barriers.

Furthermore, a stage-aligned graded exposure strategy for night shift scheduling may be explored as a potential practice implication derived from the study findings, rather than an evidence-established intervention, as this model has not been empirically tested in the present study. Specifically, interns may be exempt from independent night shifts during the Shock Period and follow a progressive sequence of day shift orientation, evening shift shadowing and half-shift assistance under appropriate clinical supervision. During the Adaptation Period, night shift duties may be escalated incrementally from assisted shifts to progressively increasing participation and responsibility, in accordance with institutional policies and training standards. In the Differentiation Period, standard departmental night shift schedules may be applied with flexible adjustment options for interns preparing for exams or recruitment. Clinical settings should adapt this framework to local regulatory requirements and staffing structures.

### 4.3 Building dynamic rotation adaptation and psychological early-warning mechanisms

The adaptation stage presents a paradoxical stress profile. On one hand, undergraduate nursing interns gradually acquire proficiency in basic nursing skills, leading to emotional stabilization and an emerging sense of achievement. On the other hand, early signs of burnout, multi-task conflicts, and variability across departments and supervisors become new stressors. From the lens of Meleis’ Transition Theory, this evolving and paradoxical pattern reflects the temporality property of transition, meaning stress responses shift systematically over time as interns progress from role insufficiency toward role mastery.

A key latent risk during this stage is early-stage burnout driven by repetitive work. When undergraduate nursing interns transition from “unable to perform tasks” to “only performing basic tasks,” a lack of new learning opportunities may significantly reduce motivation and professional engagement (21). At the same time, multi-department rotation, while a source of stress, can also serve as a resource for mitigating burnout if appropriately structured (22). Therefore, hospitals should design progressive learning pathways within rotation plans. After mastering basic skills, undergraduate nursing interns should be gradually involved in more cognitively demanding tasks such as nursing rounds, patient monitoring documentation, and preoperative education. In this way, rotations can represent not only changes in setting but also expansions in competency.

Another critical issue is the variability in departmental environments and teaching styles, which leads to highly individualized and context-dependent stress experiences. Current feedback mechanisms in many teaching hospitals rely on end-of-rotation evaluations, which are often delayed and fail to provide timely support (23).

Based on the high-risk touchpoints identified through journey mapping, embedding psychological early-warning systems into the rotation process could be explored as a potential practice direction. Regular, touchpoint-based psychological screening may be adopted to track interns’ emotional status across rotations. When persistent negative emotions related to teaching relationships or workload are identified, early support measures such as rotation adjustments or psychological counseling may be offered to prevent the accumulation of negative experiences from evolving into professional detachment in later stages. This proactive monitoring model aligns with a student-centered approach and may enable more targeted support.

Additionally, the adaptation stage often coincides with peak periods of competing demands, including postgraduate exam preparation, thesis writing, and clinical responsibilities. Under such pressure, undergraduate nursing interns face not only time constraints but also emotional burdens such as guilt and helplessness due to role conflict (24). Hospitals may consider proactively identifying peak conflict periods and providing flexible scheduling where clinically feasible. Institutions may also explore embedded micro-courses focusing on efficient multi-tasking strategies, to help undergraduate nursing interns optimize productivity within fixed time constraints. Such systemic support may help maintain stress within a manageable range without undermining professional identity.

### 4.4 Improving lifecycle career development and social support systems

The differentiation stage represents the second peak of internship stress, during which stressors shift from task-related to development-related concerns, particularly employment and further education. Aligned with Meleis’ Transition Theory, this second stress peak and the accompanying identity-related challenges are closely associated with the concept of disconnectedness, which is a core destabilizing response during transitional change. Interns in this phase experience dual forms of disconnectedness: they gradually disconnect from their former student identity as they navigate career decision-making and approach full professional roles, and they also experience social disconnectedness from family support systems, as family members’ lack of understanding of clinical contexts limits emotional empathy and weakens support effectiveness. In the Chinese context, the two core development pathways driving these concerns are civil service recruitment examinations for stable public-sector employment and national postgraduate entrance examinations for academic advancement. Both are characterized by extremely competitive selection rates, and their preparation and examination schedules directly overlap with the final months of clinical internship. The combination of this dual disconnectedness and the high-stakes, time-conflicting nature of these career pathways explains why development-related concerns become the dominant stressor in this stage, which is further distinguished by polarization of professional identity, consolidation of burnout, and weakening of support systems.

Previous studies have focused primarily on career outcomes at the end of internships, while overlooking the process through which professional identity becomes polarized. This study suggests that such divergence is not the result of a single event, but rather the cumulative outcome of stress experiences and emotional feedback throughout the internship. Sustained positive reinforcement and strong support systems enhance professional identity (25), whereas prolonged negative experiences, compounded pressures, and lack of support may lead to loss of professional meaning and eventual career withdrawal (26). Moreover, this study identified a decline in the effectiveness of support systems during this stage. When undergraduate nursing interns seek support from family members, a lack of understanding of clinical contexts often limits emotional empathy, thereby intensifying stress (27).

From a positive psychological functioning perspective, positive evaluations of the self and one’s life are consistently associated with higher self-esteem and greater life satisfaction (28). These positive psychological dispositions may act as potential protective resources for nursing interns: interns with higher levels of positive self-evaluation may be more likely to interpret challenging clinical experiences as growth opportunities rather than overwhelming threats, which could in turn support the consolidation of positive professional identity and mitigate the risk of career withdrawal.

Targeted to these challenges, clinical teaching teams may consider aligning support initiatives with undergraduate nursing interns’ lived experiences to reconstruct professional meaning. This could be explored through structured initiatives such as sharing pathways in specialty nursing development and facilitating career trajectory dialogues with exemplary nurses, thereby strengthening interns’ recognition of professional value and mitigating the erosion of meaning. Hospitals may integrate career development guidance across the entire internship trajectory, with phase-specific support during the differentiation stage. These may include tailored employment guidance, structured interpretation of residency training policies, and preparation for postgraduate re-examinations. In parallel, coordination with clinical training sites may help ensure protected time and logistical support for job seeking and further education processes. Moreover, a tripartite collaborative model involving schools, clinical institutions, and families could be explored. Providing appropriate orientation and guidance to family members may enhance their capacity for emotional support and help compensate for the support gap commonly observed in the later stages of internship.

For undergraduate nursing interns experiencing entrenched burnout or expressing strong intentions to leave the profession, individualized career counseling and psychological support may be provided. Such support measures should balance respect for personal career choices with efforts to identify and cultivate opportunities for professional development, thereby providing comprehensive and flexible reference for diverse career trajectories.

### 4.5 Limitations

Participants were recruited from five tertiary hospitals in Sichuan Province, which may limit the generalizability of the findings to other regions, secondary hospitals and community-based healthcare settings where clinical workload and teaching resources vary significantly. Future research is needed to validate the transferability of the findings of this study across diverse clinical and educational contexts.

## 5 Conclusion

Using a journey mapping approach, this study constructed a four-stage trajectory of internship-related stress among undergraduate nursing interns. The findings confirm the inherently dynamic nature of internship stress, with dominant stressors shifting systematically across phases: the preparation period is marked by anticipatory anxiety driven by information asymmetry; the shock period centers on task-related stress from competence gaps and role mismatch; the adaptation period features burnout from repetitive work and multi-role conflicts; and the differentiation period is dominated by development-related stress around career decision-making and professional identity reconfiguration. This three-dimensional journey map integrating tasks, emotional experiences and pain points provides an empirical basis for nursing educators and clinical managers to optimize internship support systems and design phase-specific support strategies. Notably, the proposed journey map requires further validation, and its transferability across diverse clinical settings, educational contexts and nursing student populations warrants further investigation.

## Data Availability

The original contributions presented in the study are included in the article/Supplementary material, further inquiries can be directed to the corresponding authors.
