# Supplementary material for: A journey mapping study of internship stress among undergraduate nursing interns
Source: Front Public Health. 2026 Oct 28;14:1958747. doi: 10.3389/fpubh.2026.1958747 (PMC13751949; doi:10.3389/fpubh.2026.1958747)
Supplement: Supplementary file 1 [file Table_1.DOCX]

**Additional file 1. English version of the study-developed semi-structured interview guide**

# Semi-Structured Interview Guide

**Introduction**

# This interview guide was developed specifically for this longitudinal qualitative study. The purpose of the interviews was to explore the dynamic process of internship-related stress among undergraduate nursing interns across different stages of clinical placement, to identify stage-specific stressors, emotional experiences, pain points, and support needs, and to inform the construction of an internship stress journey map.

**Demographic questions:** Age, Gender, Affiliated teaching hospital, Only child status, Family members working in healthcare-related fields, Career intention.

**Interview questions：**

# ****Baseline Survey****

# 1.Could you tell me about the basic information regarding your internship arrangements?

# 2.What are your overall perceptions of the upcoming clinical internship?

# 3.What are your primary sources of stress?

# 4.What areas do you perceive as insufficiently prepared?

# 5.What are your current coping strategies?

# 6.What support needs do you expect prior to the internship?

**Subsequent Follow-up**

# 1.What are your recent internship experiences?

# 2.What are your current stressors and where do they come from?

# 3.How has your stress changed compared with the previous stage?

# 4.What significant events have happened recently, and how have they affected your stress?

# 5.What is your current emotional state?

# 6.What coping strategies are you using, and have they changed over time?

# 7.How adequate do you perceive the support you receive, and what barriers do you face when seeking help?

# 8.How do you manage your time and energy across competing tasks?

# 9.Have there been any turning points related to adaptation or stress reduction? If so, what were they?

# 10.How have your current experiences influenced your career choices and professional identity?
